# Incorporating causality in energy consumption forecasting using deep neural networks

**DOI:** 10.1007/s10479-022-04857-3

**Published:** 2022-07-30

**Authors:** Kshitij Sharma, Yogesh K. Dwivedi, Bhimaraya Metri

**Affiliations:** 1 Department of Computer Science, Norwegian University of Science and Technology, Trondheim, Norway; 2 Emerging Markets Research Centre (EMaRC), School of Management, Swansea University, Room #323, Bay Campus, Fabian Bay, Swansea, SA1 8EN Wales, UK; 3 Department of Management, Symbiosis Institute of Business Management, Pune & Symbiosis International (Deemed University), Pune, Maharashtra India; 4 Indian Institute of Management Nagpur, Nagpur, India

**Keywords:** Deep neural networks, Energy consumption, Forecasting, Machine learning

## Abstract

Forecasting energy demand has been a critical process in various decision support systems regarding consumption planning, distribution strategies, and energy policies. Traditionally, forecasting energy consumption or demand methods included trend analyses, regression, and auto-regression. With advancements in machine learning methods, algorithms such as support vector machines, artificial neural networks, and random forests became prevalent. In recent times, with an unprecedented improvement in computing capabilities, deep learning algorithms are increasingly used to forecast energy consumption/demand. In this contribution, a relatively novel approach is employed to use long-term memory. Weather data was used to forecast the energy consumption from three datasets, with an additional piece of information in the deep learning architecture. This additional information carries the causal relationships between the weather indicators and energy consumption. This architecture with the causal information is termed as entangled long short term memory. The results show that the entangled long short term memory outperforms the state-of-the-art deep learning architecture (bidirectional long short term memory). The theoretical and practical implications of these results are discussed in terms of decision-making and energy management systems.

## Introduction

Time-series forecasting is one of the most important quantitative models in which historical observations of the same variable are collected and analysed to develop a model that captures the underlying data generating process. Then the model is used to predict the future. In operation research (OR), such methods are used for volatility prediction (Vidal & Kristjanpoller, 2020), risk assessment (Du et al., 2020), supply chain management (Pacella & Papadia, 2021), price forecasting (Demir et al., 2021), demand and supply forecasting (Chen et al., 2020; Chen & Lu, 2021; Du et al., 2021; van Steenbergen & Mes, 2020; Zhang et al., 2021) and so on. There are multiple methods used in time series analysis that can be divided into three broad categories: 1) classical statistical modelling and forecasting (e.g., ARIMA, GARCH); 2) machine learning and deep learning; and 3) hybrid approaches. This paper used a novel method called "Entangled Recurrent Neural Network" (E-RNNs) for time series prediction (proposed by Yoon & van der Schaar, 2017). Thus, contributing to the deep learning-based methods for time series prediction in OR. As an application of the method, this paper forecasts the energy demands in the different regions.

Energy demand forecasting is becoming increasingly crucial for planning for energy consumption, formulation of distribution strategies, and recommending modern energy policies (Bhattacharyya & Timilsina, 2009). Energy is one of the most vital resources for developing and sustaining that development in any country (Suganthi & Samuel, 2012). This is true for all the sectors that are social, economic, and environmental. Energy is also a crucial part of multiple industries such as production, agriculture, health, and education. Therefore, it is important to have an efficient energy demand management system for allocating the available resources properly and effectively. Energy demand and supply can also be seen as the macro supply chain operations, with the energy companies and distributors as the main body. For example, Bagchi et al. (2022) showed how energy usage could be employed to achieve a better energy mix to, in turn, obtain the much-desired decoupling of growth and sustainable development in developing countries. Moreover, because of the widespread impact of energy consumption, the decision-making in this sector might involve multiple stakeholders, a multitude of uncertainty, and risk inflicting sources (Dorsman et al., 2021). The decision-making in the energy sector can also be different based on the time frame (long, medium, and short term) and area scopes (country, region, city, buildings). Such decisions can also affect the financial aspects of the organizations because the energy investments have capital intensive nature. Furthermore, with the increasing world population (that is estimated at 8.5 billion by 2030; United Nations, 2015), the energy demand will also increase. This increase in demand can only be met by using efficient energy planning and sustainable energy policies, both of which depend on a reliable energy demand forecasting system. Energy demand and supply can also be seen as the macro supply chain operations, with the energy companies and distributors as the main body. Therefore, accurate forecasting of the energy demand does not only have societal impacts but also can have financial impacts. Therefore, accurate forecasting of energy demand or consumption is the desired outcome of the applied methods. One of the most prominent states of the art methods of forecasting the energy demand is using recurrent neural networks.

Recurrent neural networks (RNNs) are the most used deep architectures for solving time-series-based prediction and/or regression tasks. They have been applied in a variety of domains. For example, speech recognition (Zhang et al., 2020), traffic forecasting (Zhang et al., 2020), language translation (Vathsala & Holi, 2020), and risk scoring (Clements et al., 2020). There are two main variants of RNNs used in the state-of-the-art research, such as standard RNNs (Rumelhart et al., 1988) and bi-directional RNNs (Schuster & Paliwal, 1997). Standard RNNs can propagate the error in only one direction and, therefore, cannot use future inputs for current prediction. On the other hand, using causal predictions with bi-directional RNNs converts them into standard RNNs (Yoon & van der Schaar, 2017). Yoon and van der Schaar (2017) addressed this limitation of Bi-directional RNNs for causal prediction by proposing a novel RNN architecture called Entangled RNN (E-RNN). By stacking an additional forward hidden layer on top of the Bi-RNN structure, the causal prediction of E-RNN is dependent on all the previous backward hidden states. E-RNN can be used in a plethora of applications, ranging from medicine to finance. Importantly, E-RNN can be combined with various state-of-the-art RNN techniques such as multilayer (Parlos et al., 1994), dropout (Srivastava et al., 2014), LSTM (Hochreiter & Schmidhuber, 1997), and GRU (Chung et al., 2015) and leads to performance gains, without the need for any additional assumptions. Entangled Recurrent Neural Networks have been used in multiple domains such as face image sequencing (Oh et al., 2021), load forecasting (Sriram et al., 2018), and other power system applications (Sriram, 2020). However, as aforementioned, to the best of the authors’ knowledge, E-RNNs have not been used in the domain of OR, especially in energy demand forecasting.

In this contribution, the relation between the weather conditions that are already used for forecasting purposes in energy consumption is used (Ghalehkhondabi et al., 2017). The causal link between energy consumption and weather conditions might improve upon the state-of-the-art forecasting accuracies. Once the demand forecasting based on the proposed causal relation and using E-RNNs is obtained, the E-RNNs’ performance is compared against the other state-of-the-art RNN techniques such as multilayer multivariate bi-bidirectional LSTM (Chung et al., 2015; Hochreiter & Schmidhuber, 1997; Parlos et al., 1994; Srivastava et al., 2014). Specifically, this paper addresses the following research question:

**How much does the causal information between the weather conditions and the energy demand improves the energy demand forecasting using deep learning networks?**

To address the aforementioned research question, the proposed method is applied to three datasets where additional information about the causal relations is used. These causal relations are between the weather conditions and the energy consumption in the training phase of the algorithm to forecast the energy consumption. These results will be compared against a multivariate bidirectional LSTM using just the weather conditions to forecast the energy consumption. Such a comparison will establish the role of causal relations in the forecasting of energy consumption. The current research in this field supports the use of deep learning methods for forecasting, but there is little to no knowledge about the role played by the factors causing the energy use in the different geopolitical landscapes such as a whole country, a region within a country, cities, and a standalone building. The causal relation also changes based on how wide the area has been considered for exploration. Therefore, it is necessary to study and understand the importance of the causal relationship between weather factors and energy consumption, especially when it comes to time-series prediction and forecasting.

The remainder of the paper is organized as follows. The second section presents an overview of deep learning applications in OR, the role of energy demand forecasting in OR, and deep learning applications for energy forecasting. The third section presents the methodology, including the description of the datasets brief introduction to LSTMs, and the proposed method. The fourth section presents the results, and the fifth section discusses the results and presents the implications. Finally, the sixth section concludes the paper.

## Related work

### Deep learning applications in operation research

Over the past few years, innovations in business analytics and operations management are becoming necessary factors for the success of the ventures (Lim et al., 2013; Mortenson et al., 2015; Ranyard et al., 2015). During these years, deep learning has been used in a variety of operations research applications. For example, supply chain management (Kilimci et al., 2019; Pacella & Papadia, 2021; Punia et al., 2020), understanding/predicting financial risk behaviour (Geng et al., 2015; Kim et al., 2020a, 2020b; Xu & He, 2020) price and price movement forecasting (Sen & Mehtab, 2021; Shahi et al., 2020), fault diagnosis (Kumar et al., 2016), maintenance prediction (Kumar et al., 2018) and asset management for maintenance prediction (Chen et al., 2021) and sustainability performance prediction (Rajesh, 2020). In the following paragraphs, a brief overview is provided about each of these sub-fields and how deep learning methods are applied within the sub-fields.

Organizations use supply chain forecasting as part of their supply chain management to fulfil the requirements of short-term and long-run aggregate forecasting. Such forecasting aids in the decision-making at strategic and tactical levels. In recent times applications of and improvements upon existing deep learning networks have been frequently utilized for this purpose. For example, Kilimci et al. (2019) used a decision integration strategy empowered using a combination of support vector regression and a deep learning network to forecast the demand in the Turkish market. Pacella and Papadia (2021) used LSTMs to forecast the demand of products in the supply chain to form a better and more accurate basis for the respective replenishment systems. Xu and He (2020) used a deep belief network to forecast the financial credit risk, as a crucial part of the supply chain finance, to maintain the sustainable profit growth for financial organizations. Along with demand forecasting, sales prediction is also important in the supply chain management to make sure that the stores do not sell overstock, avoid understocking, reduce losses and minimize waste. In this vein, Husna et al. (2021) used LSTM and convolutional neural networks to forecast the sales of grocery stores. Other examples of sales forecasting in the different sectors include fashion retail (Giri et al., 2019; Loureiro et al., 2018), e-commerce (Pan & Zhou, 2020; Qi et al., 2019), pharmaceuticals (Chang et al., 2017; Ferreira et al., 2018) to mention a few.

There are several examples where improvements upon existing deep learning methods were proposed. For example, Punia et al. (2020) proposed a novel and improved cross-temporal deep learning architecture to forecast all levels (e.g., individual stock units, product groups, online and offline channels) of a retail supply chain. In another example, Kegenbekov and Jackson (2021) used an adaptive deep reinforcement learning method to achieve synchronization between the inbound and outbound flows in an organization. In all these examples mentioned above, the models incorporating deep learning methods outperform various traditional methods. These traditional methods include basic machine learning algorithms (Husna et al., 2021; Kilimci et al., 2019; Xu & He, 2020), statistical methods (Pacella & Papadia, 2021; Xu & He, 2020), and sometimes even pre-existing deep learning methods (Punia et al., 2020). Another facet of deep learning applications for supply chain management is for verifying, generating, and augmenting supply chain maps. For this purpose, Wichmann et al. (2020) used natural language processing and LSTM to automatically extract the buyer–seller relations from texts and then use the features to generate basic supply chain maps and verify/augment existing ones. More recently, Guan and Yu (2021) used the features from the resource distribution allocation index and a deep learning network to inform the design of the supply chain resource distribution allocation model.

For predicting financial risk behaviour, deep learning has been used at various stages of an artificial intelligence pipeline. In other words, deep learning has been used not only at the prediction stage of a process but also at earlier stages as well, such as during the feature extraction and selection phases. For example, Kim et al., (2020a, 2020b) used deep learning to extract features from structured data for retail traders to identify/predict risk-related behaviour. Kim et al., (2020a, 2020b) showed that the features extracted using deep learning methods provided better prediction outcomes than the traditional methods. Similarly, Geng et al. (2015) used deep neural networks to extract features from the data collected about customers to predict the bankruptcy chances of the customers. Another financial risk venture for deep learning algorithms is in the direction of volatility. Liu (2019) showed that the LSTM-RNN-based methods outperformed one of the most popular techniques (GARCH, Pérez-Cruz et al., 2003) for volatility prediction. Similar results and trends were reported by Xiong et al. (2015) in the case of volatility prediction and comparison with GARCH. Chatzis et al. (2018) used deep learning methods to forecast the stock market crisis and showed the prevalence of such methods over the classical methods. Along with similar trends, Moews et al. (2019) reported better performances shown by deep learning models when compared to traditional machine learning models. Eachempati et al. (2021) have also shown that the deep neural networks outperform the traditional methods of predicting the accounting disclosure, another type of financial behaviour that has gained considerable attraction from the deep learning applications (Almagtome, 2021). Another aspect of risk behaviour that was detected using such techniques is the detection of fraudulent reviews for online marketing websites (Kumar et al., 2022a, 2022b). Kumar et al., (2022a, 2022b) examined the different pre-processing and feature engineering techniques to extract both reviews and review-centric features and showed that unifying these features in ML classifiers resulted in better detection of fraudulent reviews than the contemporary methods. Furthermore, using deep learning techniques and text mining Huang et al. (2022) provided a more accurate estimate of financial distress for beneficiaries and investors than classical machine learning algorithms.

Concerning the stock market prices, deep learning has attracted a multitude of efforts in the direction of stock price forecasting. To compare the different deep learning models such as LSTM and GRU, Shahi et al. (2020) showed that there was no significant difference while using the models, but when the authors added additional sentiment data to forecast the stock prices, the performance was significantly increased. The high predictive performance of deep and extreme machine learning algorithms for stock price prediction was also reported by Balaji et al. (2018), Sen and Mehtab (2021), and Liu et al. (2022). Furthermore, Wang and Fan (2021) show that by incorporating complex non-linear relations into the architecture of the deep learning networks, one can achieve high stock price prediction capacity. Sirignano and Cont (2019) used a deep temporal network and trained it on all the stock data that they obtained and showed that this model outperformed individually trained models. Li and Pan (2022) proposed an ensemble deep learning model for predicting stock prices using the current affairs of the companies. ) used a generative adversarial network for stock market price movement prediction. Another set of efforts used sentiment analysis of Twitter data for stock price prediction (Darapaneni et al., 2022; Jing et al., 2021; Mohan et al., 2019; Rao & Srivastava, 2012; Shivaprasad and Shetty, 2017; Swathi et al., 2022; Yusof et al., 2018). Sirignano and Cont (2019) further claim that the "general" model can also be used for "transfer" learning purposes. A sub-application of stock price prediction is predicting the stock price movements and price formulation. For example, Tsantekidis et al. (2017) used stochastic deep learning networks to forecast the price movement from the large-scale high-frequency data. In the same vein, Zhao and Chen (2021) integrated ARIMA, convolutional neural networks, and long-short term memory to detect non-linear temporal patterns and predict the stock price movement. For deeper reviews on the applications of the deep learning algorithms in the domain of finance, see the surveys done by Ozbayoglu et al. (2020) and Sezer et al. (2020).

### Energy demand/consumption forecasting

Energy demand forecasting/prediction is not a new problem. However, as mentioned in the introduction that it has become, over the years, an important problem to solve from an OR perspective. In the following, a few examples are described of how the past researchers have addressed the problem of energy demand/consumption forecasting/prediction. For comprehensive and in-depth reviews, see, Ghalehkhondabi et al. (2017), Islam et al. (2020), and Suganthi and Samuel (2012). The examples covered in this brief overview, methods to forecast/predict the energy demand/consumption, are from basic time-series, regression and econometric analysis, ARMIA and GARCH models, basic machine learning, and deep learning methods.

Time series models are concerned with trend analysis, Markov models, and spectrum analysis. Ediger and Tatlıdil (2002) analysed the cyclic patterns in the energy consumption data to forecast the energy demands in Turkey. Aydin (2014) used distribution analysis (t- and F-distributions) to forecast the demand for energy from fossil fuels globally. Aydin (2015) used a similar analysis to model the trends of coal-based energy demands in countries such as India, the United States, Japan, South Africa, and Thailand. Farajian et al. (2018) used the Box-Jenkins method for trend analysis to provide the agriculture energy demands in Iran over a 24-year period. Morakinyo et al. (2019) used the trend analysis to predict the energy consumption on the extreme weather days to delineate the effect of the extremely hot or extremely cold days. Tian et al. (2022) conducted a similar analysis as Morakinyo et al. (2019) with additional regional climate models to predict the energy consumption during the extreme weather days in various regions of Canada.

Efforts from a regression analysis point of view have used different variables to predict the energy demands of households, buildings, and regions. For example, Harold et al. (2017) used the income elasticity of households and quantile regressions to predict the energy consumption, with the aim of informing the use of constant mean elasticity for policy purposes. Maaouane et al. (2021) used the import, export, and energy efficiency measures information to predict the energy demands in the industrial sector. Catalina et al. (2013) used the global heat loss coefficient of buildings, the indoor set point, the sol–air temperature difference, and the south equivalent surface to predict the heating energy consumption. Many studies have considered whether factors as independent variables in regression analysis to predict the energy demands. For example, Braun et al. (2014) used humidity and temperature to predict the energy demands of supermarkets in the UK. Fumo and Biswas (2015) used indoor and outdoor temperature and solar radiation to predict the energy consumption of residential buildings. Tso and Guan (2014) also predicted the residential energy demands using the house size, housing type, heating requirement, and amount of air-conditioning use and a multiple regression model.

Econometric models use the correlations between the energy demand and the macro-economic variables to predict/forecast the energy demands/consumption. One of the methods for incorporating the macro-economic variables in the analysis is to use the causal analysis. For example, Kayhan et al. (2010) used the causality between economic growth and energy consumption to forecast the energy demands in Romanian households. In another study, Sentürk and Sataf (2015) used the GDP and other socio-econometric information from the World Economic Forum for seven countries (Turkey, Kazakhstan, Azerbaijan, Kyrgyzstan, Uzbekistan, Turkmenistan, and Tajikistan) to predict the overall energy consumption in those countries. Ozturk et al. (2010) used similar information to analyse the causality between the GDP and the energy consumption for 51 countries and showed that for the low-income counties, the GDP Granger caused the energy consumption; while for the middle-income countries, there was a bi-directional causality. Other examples of studies using economic growth to cointegrate/predict energy consumption at a large scale could be found in Sentürk and Sataf (2015).

The next category of tools used is the models with auto-regressive components, that is, auto-regressive moving average (ARMA), auto-regressive integrated moving average (ARIMA), and generalized autoregressive conditional heteroskedasticity (GARCH). ARIMA and/or ARMA models are used to extract the historical trends in the time-series data and use this information for forecasting purposes (Erdogdu, 2007; Ho & Xie, 1998; Huang & Shih, 2003; Vo et al, 2021; Wang et al., 2019). The only difference between the two models is that, unlike ARMA models, the ARIMA models can be used only with stationary time-series (Valipour et al., 2013). On the other hand, GARCH models have similar functionality as ARMA/ARIMA models with one key difference (Bauwens et al., 2006; Engle, 2001). While ARMA/ARIMA models utilize the conditional mean of the time series to forecast the values, GARCH models use the conditional variances in the time series to perform similar forecasts. These models are especially useful when there is heterogeneity in the time series (Bauwens et al., 2006; Engle, 2001). Examples of studies using ARMA/ARIMA models for predicting the future energy demands include Li and Li (2017) predicting the future energy consumption in the Shandong province in China; Ozturk and Ozturk (2018) predicting the coal, oil, natural gas, and renewable energy consumption in Turkey. Furthermore, Eerdogdu (2007) also used cointegratison analysis with ARIMA to predict total energy consumption in Turkey, while ) used ARIMA models to predict agricultural loads at small scales. Other similar efforts include predicting energy consumption in Morocco (Kafazi et al., 2016), Ghana (Sarkodie, 2017), Afghanistan (Mitkov et al., 2019), India, China, and the USA (Jiang et al., 2018), and Middle Africa (Wang et al., 2018). In the case of using GARCH for prediction purposes in the energy sector, the primary use cases of this method if predicting the volatility of the energy market and load forecasting. For example, Efimova and Serletis (2014) use the extreme weather conditions, geopolitical tensions, and de-regularised markets along with GARCH models to forecast the volatility in the energy market; while Ergen and Rizvanoghlu (2016) used GARCH and the historical changes due to weather and demand abnormalities to forecast the volatility in the natural gas energy demand. Fałdziński et al. (2020) used GARCH and SVM to forecast the volatility in the demands of multiple energy sources (e.g., oil, gas, gasoline). Concerning load forecasting, Hor et al. (2006) used GARCH not only for the load forecast daily but also to estimate the maximum daily demand with high accuracy. Similarly, Iwafune et al. (2014) used GARCH to forecast a building's energy load in short-term usage. For a comparison of time-series-based methods for energy demand forecasting, see Okawu et al. (2019).

Another category of studies to forecast the energy demands is concerned with the basic machine learning algorithms. For example, Eseye and Lehtonen (2020) used Artificial Neural Networks (ANN) and Support Vector Machines (SVM) with the weather, occupancy, and heat requirement data, to predict the energy consumption for residential buildings in Finland. Pelka (2021) also used SVM and ARIMA models to predict the mid-term energy consumption in European countries. Wu and Shen (2018) use a swarm optimization-based SVM to predict the natural gas consumption. Johannesen et al. (2019) compared different machine learning algorithms for different time units and concluded that random forest regressors were the best performing for the long-term forecasting problems, while K-Nearest neighbour based regressors were the most efficient for the short term forecasts. Ahmad and Chen (2019) used Random Forest with non-linear auto-regression techniques to predict the energy demands during the different seasons and with the aim of having a more accurate grid-based distribution scheme than before. Wang et al. (2018) also used Random Forests to predict short-term energy demands for building in Florida.) compared future energy predictions using the ARIMA and Random Forest models, and the results show that Random forest was better performing for the long-term prediction between the two models. Other examples of using basic machine learning algorithms include Murat and Ceylan (2006) using ANN to predict the energy demand in the transport sector; Kankal and Uzlu (2017) also using ANN for long-term energy demand forecast in Turkey; Ferlito et al. (2015) using ANN to forecast a building's energy consumption; Lu et al. (2021) using SVM for forecasting the energy consumption in the USA; Ghazal et al. (2022) using IoT data and fusion of SVM algorithms to predict the industrial energy consumption and; Jana and Ghosh (2022) using discrete wavelet transform and ensemble machine learning algorithms to forecast natural gas prices and demand. Forouzandeh et al. (2022) also used ensemble machine learning algorithms to predict room energy demand. For a comprehensive review of studies using artificial neural networks and support vector machines for energy demand forecasting, see Ahmad et al. (2014).

Finally, the most recent set of studies (as is clear from the advancement of the methods and computing power in the last decade) concern the methods related to deep learning for forecasting the energy requirements in various sectors (e.g., household, industries), at various time scales (e.g., short-term, mid-term, long-term), and for the different area-scope (e.g., buildings, regions, countries). For example, Hrnjica and Mehr (2020) used the time-series decomposition and Recurrent Neural Networks (RNN) to predict the energy demand in Northern Nicosia, Cyprus. Real et al. (2020) used a combination of Convolutional Neural Networks (CNN) and ANN for load forecasting in French grids. Ishaq and Kwon (2021) used an Ensemble of deep network architectures to forecast short-term energy demands for local Korean buildings. Somu and Ramamritham (2021) also predicted the future energy for a local building using combinations of CNN and LSTM. Al Khafaf et al. (2019) used LSTM to forecast energy demands in Victoria, Australia. Kim and Cho (2019a, 2019b) also used LSTM and an auto-encounter for short-term forecasting of power demands in households. There have been a few studies where the traditional machine learning algorithm and deep learning algorithms were compared in terms of their forecasting accuracies. For example, Paterakis et al. (2017) compared multiple layer perceptron against Random forest, SVM, and other regressors; Ağbulut (2022) compared deep neural networks against SVM to predict the energy demands for the transport sector; Bakay and Ağbulut (2021) compared deep neural networks against SVM and ANN to forecast electricity demands in Turkey; Shirzadi et al. (2021) compared LSTMs against SVM and random forest to forecast long-term power demands in Ontario, Canada. In all these examples, the deep learning algorithms outperformed the basic machine learning algorithms. Another example of using deep networks in the energy sector is to perform the assets management for the electrical grid companies (Kala et al., 2020), where the authors showed that their proposed algorithm involves the faster regional convolutional neural networks outperformed the human-coding efforts for asset management. For a comprehensive review of deep learning for energy systems and building energy, please see, Forootan et al. (2022) and Ardabili et al. (2022), respectively. From the studies reported in this section, there is a lot of potential in using deep learning methods to forecast energy consumption as a process in Operations Research. This contribution aims to improve upon the existing deep learning algorithms to predict future energy demands/consumption by incorporating the causal relation between the weather information and the energy consumption over different area-scope (e.g., buildings, regions, countries).

## Methodology

### Granger causality

Granger causality (Granger, 1969) tests for the ability of one time series to predict another one – in the present case, whether information flow provides sufficient information to predict 1) user focus size, 2) cognitive load, and 3) user attention flow. Granger causality investigates bi-directional, simultaneous, and continuous relationships and has been employed in several studies in OR (e.g., Ghouali et al., 2014; Mian & Liang, 2010; Tang & Chrsquo, 2011; Zhang & Xu, 2015). The basic definition of Granger causality has two assumptions (Granger, 1969). First, it assumes that the cause occurs prior to the effect. Second, the cause contains information about the effect that is more important than the history of the effect itself. Although Granger causality is defined for linear and stationary time-series contexts, variations for non-linear (Ancona et al., 2004; Chen et al., 2004) and non-stationary (Ding et al., 2000; Hesse et al., 2003) data exist.

The main idea behind Granger’s definition of causality is that if the lag (past values) of variable one predicts the current value of variable two in a better manner than the lags (past values) of the variable two itself, it can be inferred that variable one causes variable two. To arrive at such an inference, there is a simple method to be followed. Considering the case of two variables, *X* and *Y*. To determine whether *X* Granger causes *Y* or the other way around, two models are created. The first model predicts the current value of *Y* using the past values of *Y*, while the second model predicts the current value of *Y* using the past values of *X*. Then, the quality of the prediction for both models is compared; if the second model outperforms the first model, it can be inferred that *X* Granger causes *Y*.

To conduct the data analysis, there are a number of statistical steps. First is data treatment, which is to divide the dataset comprising of weather information and energy consumption into 48 h windows for further analyses. Then the stationary nature of the time series is tested: a Ljung-Box test is used to determine whether there are significant non-zero correlation coefficients at lags 1–15. Small p-values suggest that the time series data is stationary. Further, the optimum value for the ‘lag’ is identified as the number of previous data points considered for modelling the causality. The value is identified based on the Akaike information criterion (AIC) value of the model. Different models are created with different values of lag that must be considered for the Granger causality consideration and select the model with the lowest AIC value.

Next, Granger Causality (Granger, 1969) is tested to examine the causality between the different variable pairs (humidity – energy consumption; information flow – energy consumption; information flow – energy consumption; wind speed – energy consumption). As aforementioned, the basic principle of Granger causality is to compare two models to test whether *x causes y*. The first model predicts the value of *y* at time *t* using the previous *n* values of *y*. The second model predicts the value of *y* at time *t* using the previous *n* values of both *x* and *y*. The comparison of the two models can tell whether the history of *x* contains more information about *y* than the history of *y* itself. If this is the case, then it can be said that *x Granger causes y*.

$$ \begin{aligned} & y(t) = \sum\limits_{j = 1}^{p} {\alpha_{11j} x(t - j) + \alpha_{12j} y(t - j) + \varepsilon_{1} t} \\ & y(t) = \sum\limits_{j = 1}^{p} {\alpha_{22j} y(t - j) + \varepsilon_{2} t} \\ \end{aligned} $$

where *p* model order, maximum lag included in the model, *α* coefficients matrix, the contribution of each lag value to the predicted value, ε residual, prediction error.

One might argue about the choice of the method to analyse the causality between the different pairs of measurements. This paper uses the definition of causality provided by Granger. There are three other methods that could be used to show the causality between different variables: 1) Structured Equation Modelling (SEM, (Edwards & Bagozzi, 2000)) 2) Cross-convergent mapping (CCM, (Sugihara et al., 2012)) and 3) conducting an intervention experiment where the hypothesized cause is controlled and the hypothesized 'effect' is measured (Shadish et al., 2002). SEM does not necessarily contain the information required to consider a causal relationship. Statistically speaking, testing an SEM is not a test for causality. There is a certain mathematical formulation under which SEM can be used for causal inference (Steyer, 2013; Steyer et al., 2002); however, the solutions are not available commercially. Bollen and Pearl (2013) provide a detailed account describing how SEM should not be used for modelling causal relations between variables. The second method, that is, CCM, is useful only in the cases where the time series is stationary (i.e., the mean and variance of the variable do not change over time) and non-linear (i.e., there is no autocorrelation in the time series). Eye-tracking data is stationary (as revealed by the Ljung-Box test) but auto-correlated (where users look at current time instances vastly depending on where they were looking at previous instances). Therefore CCM is not an adequate method for such data. In the case of identifying causal relations between two variables through an experimental or pseudo-experimental setup, such setups are typically costly or require an extensive duration to identify the cause-effect relationship between the two variables in question (Chambliss & Schutt, 2018). Moreover, it has also been shown that for longer time-series data, the Granger causality outperforms other contemporary methods (Zou & Feng, 2009).

In this contribution, four casualties for each dataset (and sub-datasets) are computed for a time window of 48 h with a one-hour shift between two consecutive windows: (1) pressure "Granger causing" demand; (2) wind speed "Granger causing" demand; (3) temperature "Granger causing" demand, and (4) humidity "Granger causing" demand. Once the F-values for all four causal relations are available over time, this is used as additional information for forecasting using entangled LSTM.

### Entangled LSTM

#### LSTM

A single LSTM cell is comprised of four components:

1. Forget gate (f): this is a neural network with a sigmoid activation function. This gate is responsible for what information is propagated to the next time step and what information is discarded. Depending on the previously hidden state h_t-1_ and the current input x_t_, the forget gate assigns a value between zero and one to every element in the previous cell state C_t-1_. For all the elements that are assigned a value of one, the information is retained, and for all the elements that are assigned a value of zero are discarded. For all the elements that are assigned a value between zero and one, the value decides how much information is to be retained.
2. Input gate (I): this is also a neural network that uses a sigmoid activation function. To make the decision about what new information is to be stored in the cell state (explained next), there are two different operations:
  - The input gate decides which values will be updated.
  - Using a tanh activation function, a set of candidate values is created ($\widetilde{{C_{t} }}$). Once $\widetilde{{C_{t} }}$ and I_t_ are computed, the input given to the cell state can be decided.
3. Cell State (C_t_): this functions as the memory of the LSTM. Due to the cell states, LSTMs usually outperform basic recurrent neural networks. For every time window, the C_t-1_ is combined with the forget gate, and it is determined what information is propagated to the next time step and what information is discarded. The retained information is then combined with I_t_ and $\widetilde{{C_{t} }}$ to create the new cell state that will be the new memory of the LSTM.
4. Output gate (O): this is another neural network that uses a sigmoid activation function. The cell state computed from the previous step is passed through a hyperbolic function (tanh), and this creates the cell values that are filtered between -1 and 1.

**Fig. 1:**
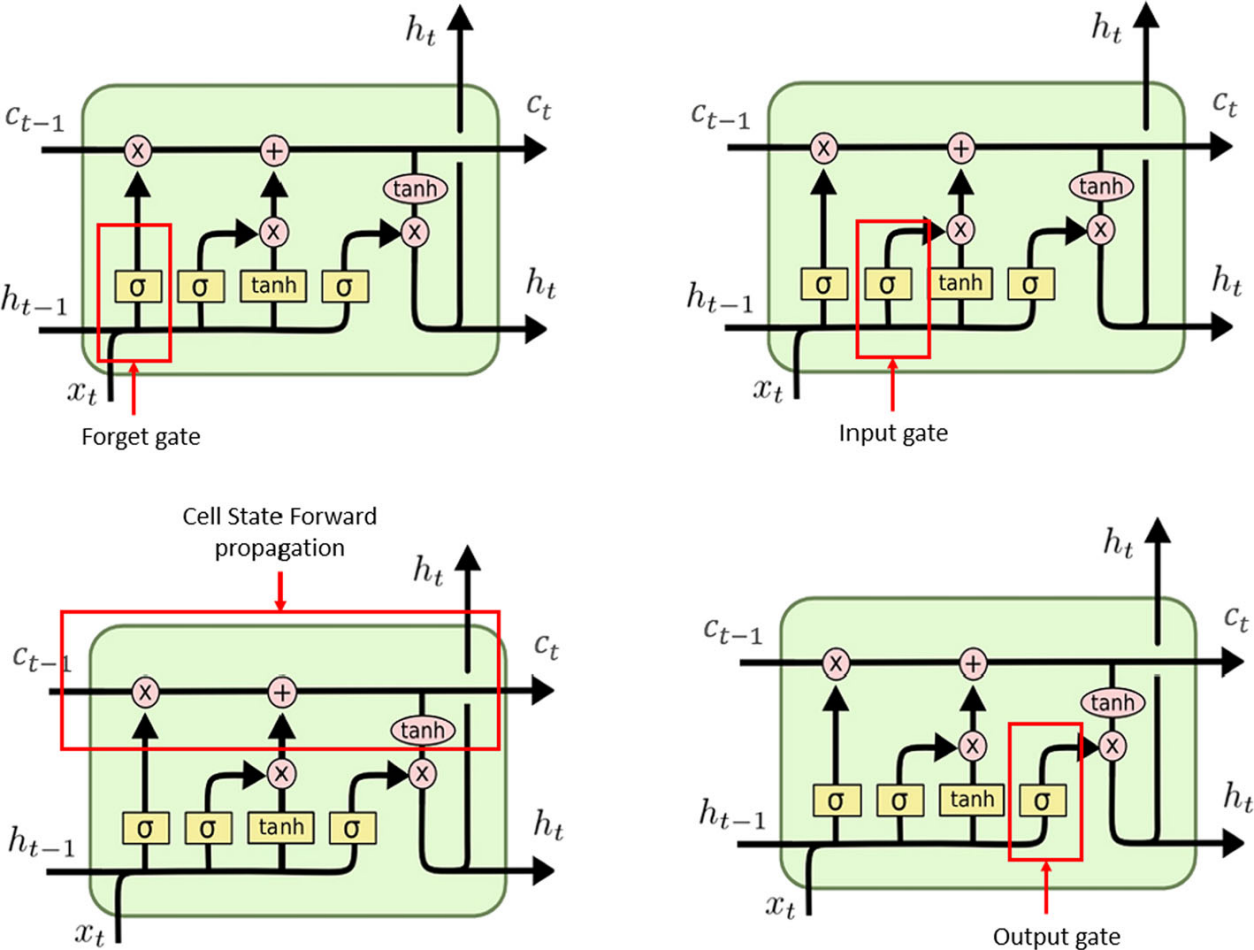
One LSTM cell with all the components marked with red boxes. (Color figure online)

The schema in Fig. 1 shows the various gates and their arrangement;

For each gate, the following are the variables (set of weights and biases):

1. W_f_ and b_f_ are the Forget gate weight and bias, respectively.
2. W_i_ and b_i_ are the Input gate weight and bias, respectively.
3. W_c_ and b_c_ are the Candidate cell state weight and bias, respectively.
4. W_o_ and b_o_ are the Output gate weight and bias, respectively.
5. W_v_ and b_v_ are the Weight and bias associated with the Softmax layer, respectively.
6. f_t_, i_t_, Ct, and o_t_ are the Output of the activation functions for the forget, input, cell, and output gates, respectively.
7. a_f_, a_i_, a_c_, and a_o_ are the Input to the activation functions to the forget, input, cell, and output gates, respectively.
8. CF is the cost function with respect to which the derivatives are calculated.

For each gate, the following equations show how the activation of each gate is calculated.

1. Forget gate: $$a_{f} = W_{f} .Z_{t} + b_{f} \;\;f_{t} = sigmoid\left( {a_{f} } \right)$$
2. Input gate: $$a_{i} = W_{i} .Z_{t} + b_{i} \;\;i_{t} = sigmoid\left( {a_{i} } \right)$$
3. Input gate: $$a_{c} = W_{c} .Z_{t} + b_{c} \;\; \widetilde{{C_{t} }} = tanh\left( {a_{c} } \right)$$
4. Cell state: $$C_{t} = f_{t} \otimes C_{t - 1} \oplus i_{t} \otimes \widetilde{{C_{t} }}$$
5. Hidden state: $$h_{t} = o_{t} \otimes tanh(C_{t} ) $$
6. Output Eq. 1: $$V_{t} = W_{V} .h_{t} + b_{t}$$
7. Output Eq. 2: $$\widehat{{y_{t} }} = softmax\left( {V_{t} } \right)$$
8. Output gate: $$a_{f} = W_{f} .Z_{t} + b_{f} \;\;f_{t} = sigmoid\left( {a_{f} } \right)$$

The outcome of all the gates is derived in a similar manner, and here the forget gate calculations are explained as an example. There is a fixed path from the activation of the forget gate to the cost function that is shown as the following:

$$ {\text{f}}_{{\text{t}}} \to {\text{C}}_{{\text{t}}} \to {\text{h}}_{{\text{t}}} \to {\text{CF}} $$

The following is how the derivative is calculated for the cost function with respect to the forget gate.

$$ \frac{dCF}{{df_{t} }} = \frac{dCF}{{dh_{t} }} * \frac{{dh_{t} }}{{dC_{t} }} * \frac{{dC_{t} }}{{df_{t} }} $$

All the derivatives of the cost function with respect to the cell state and hidden state are calculated in the same manner. Input to each LSTM is the previous cell state and the concatenated previous hidden state and current input. For simplicity [h_t-1_, x_t_] Z_t_

$$ \frac{dCF}{{dC_{t - 1} }} = \frac{dCF}{{dC_{t} }} * \frac{{dC_{t} }}{{dC_{t - 1} }} = \frac{dCF}{{dC_{t} }} * f_{t} $$

$$ \begin{aligned} & \frac{dCF}{{dZ_{t} }} = \frac{dCF}{{da_{f} }} * \frac{{da_{f} }}{{dZ_{t} }} + \frac{dCF}{{da_{i} }} * \frac{{da_{i} }}{{dZ_{t} }} + \frac{dCF}{{da_{o} }} * \frac{{da_{o} }}{{dZ_{t} }} + \frac{dCF}{{da_{c} }} * \frac{{da_{c} }}{{dZ_{t} }} \\ & \quad \quad \quad \quad = W_{f}^{T} * \frac{{da_{f} }}{{dZ_{t} }} + W_{i}^{T} * \frac{{da_{i} }}{{dZ_{t} }} + W_{o}^{T} * \frac{{da_{o} }}{{dZ_{t} }} + W_{c}^{T} * \frac{{da_{c} }}{{dZ_{t} }} \\ \end{aligned} $$

The forget gate:

$$ \frac{dCF}{{df_{t} }} = \frac{dCF}{{dh_{t} }} * \frac{{dh_{t} }}{{dC_{t} }} * \frac{{dC_{t} }}{{df_{t} }} $$

But,

$$ \frac{dCF}{{dC_{t} }} = \frac{dCF}{{dh_{t} }} * \frac{{dh_{t} }}{{dC_{t} }} $$

So,

$$ \frac{dCF}{{df_{t} }} = \frac{dCF}{{dC_{t} }} * \frac{{dC_{t} }}{{df_{t} }} $$

$$ \frac{dCF}{{df_{t} }} = \frac{dCF}{{dC_{t} }} * \frac{{d \left( {f_{t} \otimes C_{t - 1} \oplus i_{t} \otimes \hat{C}_{t} } \right)}}{{df_{t} }} $$

$$ \frac{dCF}{{df_{t} }} = \frac{dCF}{{dC_{t} }} * C_{t} $$

And,

$$ \frac{dCF}{{da_{f} }} = \frac{dCF}{{df_{t} }} * \frac{{df_{t} }}{{da_{f} }} $$

$$ \frac{dCF}{{da_{f} }} = \frac{dCF}{{dC_{t} }} * C_{t - 1} * f_{t} \left( {1 - f_{t} } \right) $$

The other derivatives can be computed with respect to the inputs and outputs of the cost function. For example, for the input gate,

$$ \frac{dCF}{{di_{t} }} = \frac{dCF}{{dC_{t} }} * \hat{C}_{t} $$

$$ \frac{dCF}{{da_{i} }} = \frac{dCF}{{dC_{t} }} * \hat{C}_{t} * i_{t} \left( {1 - i_{t} } \right) $$

For the Cell State:

$$ \frac{dCF}{{dC_{t} }} = \frac{dCF}{{dh_{t} }} * O_{t} * \left( {1 - tanh^{2} \left( {C_{t} } \right)} \right) $$

$$ \frac{dCF}{{d \hat{C}_{t} }} = \frac{{dC_{t} }}{{d \hat{C}_{t} }} * i_{t} $$

$$ \frac{dCF}{{da_{c} }} = \frac{dCF}{{dC_{t} }} * i_{t} * \left( {1 - \hat{C}_{t}^{2} } \right) $$

Output gate:

$$ \frac{dCF}{{dO_{t} }} = \frac{dCF}{{dh_{t} }} * tanh (C_{t} ) $$

$$ \frac{dCF}{{da_{o} }} = \frac{dCF}{{dh_{t} }} * tanh \left( {C_{t} } \right) * O_{t} \left( {1 - O_{t} } \right) $$

and finally, the hidden state:

$$ \frac{dCF}{{dh_{t} }} = \frac{dCF}{{dV_{t} }} * \frac{{d \left( {W_{V} * h_{t} } \right)}}{{dh_{t} }} = W_{V}^{T} * \frac{dCF}{{dV_{t} }} $$

The weights for all the gates.

Forget gate:

$$ \frac{dCF}{{dW_{f} }} = \frac{dCF}{{da_{i} }} * \frac{{da_{f} }}{{dW_{f} }} $$

Input gate:

$$ \frac{dCF}{{dW_{i} }} = \frac{dCF}{{da_{i} }} * \frac{{da_{i} }}{{dW_{i} }} $$

Output:

$$ \frac{dCF}{{dW_{V} }} = \frac{dCF}{{dV_{t} }} * \frac{{dV_{t} }}{{dW_{V} }} $$

Output gate:

$$ \frac{dCF}{{dW_{o} }} = \frac{dCF}{{da_{o} }} * \frac{{da_{o} }}{{dW_{o} }} $$

#### Bi-directional-LSTM

Bi-directional LSTM, or Bi-LSTM, functions on a very simple principle. In Bi-LSTMs, there are just two LSTMs put together. The first LSTM runs on the exact input sequence that is provided by the dataset. The second LSTM runs in the reversed order of the input sequence as the first LSTM. This improves the LSTM by using both the temporal directions: past and future. The forward layer is responsible for the positive time (forward states or the future), and the backward state is responsible for the negative time (backward state or the past). It has been shown in various contexts that Bi-LSTM outperforms the simple LSTM. For example, Sun et al. (2018) reported better prediction from using Bi-LSTM as compared to LSTM while predicting the blood glucose levels. Shahid et al. (2020) also reported better prediction quality from Bi-LSTM than LSTM while predicting the COVID-19 infections. In terms of energy consumption prediction, Le et al. (2019) showed better performance of Bi-LSTM than regular LSTM. Moreover, Kim and Cho (2019a, 2019b) also show better energy consumption forecasting in specific regions while using Bi-LSTMS than regular LSTMs, whereas Ma et al. (2020) showed similar results while predicting the future energy consumption of individual buildings. Other examples where Bidirectional LSTMs outperformed LSTM in time-series forecasting/prediction tasks include crop detection (Crisóstomo et al., 2020), text mining (Alzaidy et al., 2019), news classification (Li et al., 2002), human activity classification (Shrestha et al., 2020) and sequence tagging (Huang et al., 2015).

The main reason for Bi-LSTM outperforming the simple LSTM can be attributed to the fact that by using two hidden states for each time step, the information from the past and the future is preserved, which in turn provides a better approximation of the time series and encodes the contexts in a better manner than just a forward layer. Therefore, Bi-LSTMs provide better forecasting performance than regular LSTMs. One of the key operations in the Bi-LSTMs is the merging of the two layers, that is, forward and backward layers. This operation is necessary because without merging, it will not be possible to combine the outputs of these layers since they function independently of each other. There are four primary ways of merging the output of these two layers. (1) Sum: The outputs are added together. (2) Multiply: The outputs are multiplied together. (3) Concatenation: The outputs are concatenated together (the default), providing double the number of outputs to the next layer. (4) Average: The average of the outputs is taken. The sum and multiplication artificially increase the variance of the outputs, while the average reduces them. At the same time, the concatenation maintains the original variances of the outputs of the forward and backward layers. Therefore most of the contributions use the concatenation operation to merge the outputs of the forward and backward layers in the Bi-LSTMs.

#### Entangled-LSTM

**Fig. 2:**
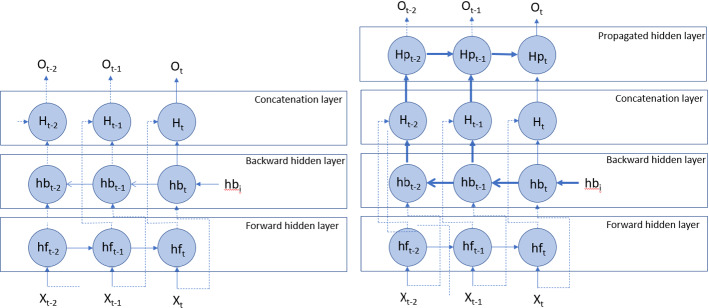
Bidirectional LSTM (left) and entangled LSTM (right). Dash line: Independent connection to o_t_, Thick blue line: New connections by Entangled-LSTM. (Color figure online)

In the Entangled-LSTM, an additional layer containing the causal information is stacked on top of the backward layer of Bi-LSTM. This layer is a traditional LSTM layer where the positive direction of time (i.e., the future) is maintained. It can be seen in Fig. 2 that the forward hidden layer that is stacked on top of the backward hidden layer is used for propagating the backward hidden state to the current output. The following shows the update process of the hidden and current output states:

$$ hf_{t} = tanh \left( {bf + Wf*hf_{t - 1} + Uf*x_{t} } \right) $$

$$ hb_{\tau } = \left\{ {\begin{array}{*{20}c} { tanh \left( {bb + Wb*hb_{i} + Ub*x_{t} } \right) it \tau = t} \\ { tanh \left( {bb + Wb*hb_{\tau + 1} + Ub*x_{\tau } } \right) otherwise} \\ \end{array} } \right. $$

$$ h_{t} = tanh \left( {b + W*h_{t - 1} + U\left[ {hf_{t} ;hb_{t} } \right]} \right) $$

$$ O_{t} = c + Vh_{t} $$

where h, hf, and HB are the hidden states in simple, forward, and backward layers, respectively; b, bf and bb are the biases in the simple, forward, and backward layers, respectively; W, Wf, Wb, U, Uf, Ub are the weights for the respective networks.

The outcome of the output layer is dependent on the propagated hidden state, the current forward and backward states. The propagated hidden state is dependent on the previous propagated hidden state, which is dependent on three states: 1) the previous propagated hidden state, 2) the previous forward hidden state, and 3) the previous backward hidden state. This chain of dependencies shows that the output at any time t is dependent on the entire input, forward, backward and hidden states. Whereas, in the Bi-LSTM, the output at time t is dependent only on the entire input and forward states only. Figure 2 shows the difference between the Bi-LSTM and entangled-LSTM schematically.

### Datasets and pre-processing

This paper used three datasets available online. Following is a brief description of the datasets and how they have been pre-processed to be used in this paper. These three datasets cover three different area scopes: the first dataset (Spain) is a country-wide dataset, the second dataset (Paraguay) is a region-specific dataset, and the third dataset (France) is for a specific building. For the convenience of expression, datasets 1, 2, and 3 are referred to as Spain, Paraguay, and France datasets, respectively, in the rest of this paper. The three datasets contain different amounts of data in terms of their time span. As reported next, the first dataset had 48 months of data, and the second and the third datasets have 3 and 29 months as the total duration of the recorded data, respectively. Therefore, for maintaining equal grounds for comparison across the three datasets, only the first 29 months of data from the first two datasets were used.

#### Dataset1: Spain

This dataset^1^ contains four years (a total of 48 months) of electrical consumption, generation, pricing, and weather data for Spain. Consumption and generation data were retrieved from ENTSOE, a public portal for Transmission Service Operator (TSO) data. Settlement prices were obtained from the Spanish TSO Red Electric España. Weather data was purchased as part of a personal project from the Open Weather API for the five largest cities in Spain and made public on Kaggle. The dataset contains the following sources of energy: biomass, coal/lignite, coal gas, natural gas, coal, oil, shale oil, peat, geothermal, hydro, sea, nuclear, and other renewable. This detailed information was not present in all the datasets. Therefore, these diverse data sources were aggregated to reflect the total demand for every hour across four years. This dataset also contains hourly weather parameters from the five largest cities in Spain that are Madrid, Barcelona, Valencia, Seville, and Bilbao. Once again, for the analysis and consistency across the datasets, humidity, temperature, pressure, and wind speed were used as the weather parameters. Further, there was no specific energy data for the separate cities; therefore, for the purpose of this contribution, the granger causality was computed (explained in the next subsection) between the weather data from all the cities separately and virtually divided this dataset into five sub-datasets, one for each city.

#### Dataset2: Paraguay

This dataset^2^ contains the electricity consumption and the meteorological data of the Alto Parana region in Paraguay. Both datasets are from January 2017 to December 2020, a total of 36 months of a time period. The weather data contains temperature (Celsius), relative humidity (percentage), wind speed (km/h, kilometres per hour), and atmospheric pressure (hPa, hectopascal) at the station level with a frequency of every three hours. To be consistent with the other two datasets, the weather data was extrapolated to represent hourly data. A simple smoothing function was used to extrapolate the weather data. The window size for the smoothing function was 24 data points (3 days) with a shift of one data point between two consecutive windows. The electricity consumption data was recorded from 55 feeders in 15 substations in an hourly fashion. Once again, to maintain consistency, the data from the 55 feeders were combined into one by aggregating the consumed amount from all the substations. This was done because there was only one weather station form where the data was gathered, and there was no specific location provided for the weather station. Another way to process this dataset was similar to dataset1, where five sets of causal relations were computed (one for each city). However, this would not have been possible here because even computing 14 causalities would be cumbersome; and because the data is from a region and not a country, therefore aggregating the electricity consumption is the better choice.

#### Dataset3: France

This dataset^3^ contains the energy consumption and weather data from one Challenger building in Guyancourt, France. The dataset has 29 months of high-frequency energy consumption data, with the recording frequency being every 10 min. The energy consumption includes Heating and cooling, electrical consumption (indoor and outdoor comfort units), Lighting, plug load, blinds, sanitary consumption, air handling unit consumption, and total consumption. For maintaining consistency across the three datasets, the total energy consumption was used, which is the aggregate of all the individual energy consumptions. The data was aggregated in terms of temporal frequency. The energy consumption data were recorded every 10 min; therefore, to compute the hourly consumption, the data from one hour (six or fewer values in certain hours) was added. The weather data included daily degrees during the days, hourly humidity, hourly temperature, and daily sunshine hours. There was no pressure or wind speed data. However, the exact coordinates of the building were available. Therefore, it was possible to extract the missing information (hourly pressure and hourly wind speed) from online resources (e.g., scrapping certain web pages and some freely available data). It was possible to extract the missing information for the whole period represented in the dataset.

### Training and testing setup

To train, validate, and test the Bi-LSTM and Entangled-LSTM, the data from the first 29 months of the three datasets were used. This was done to have an accurate comparison among the three datasets because 29 months was the lowest of the durations across them. The input of the data for the Bi-LSTM contains the batch size (48 h, i.e., two days), number of time steps (120 h, i.e., five days), and the hidden size. The input data is then fed into three "stacked" layers of LSTM cells (of 50 lengths for the hidden size), and the LSTM network is shown as unrolled over all the time steps. The term "unrolled" means that the feedback loop of an LSTM cell is not shown. The loop is used for keeping the information persisting within the recurrent network. The output from these unrolled cells is the same as the input (batch size, number of time steps, hidden size). This output data is passed to the time distributed layer, which is the set of inputs that the model will learn from to predict the input data coming after. Finally, the output layer has a softmax activation applied to it. This output is compared to the training data for each batch, and the error and gradient are then backpropagated. The Entangled LSTM is created in the same manner as the Bi-LSTM except for one difference. In Entangled-LSTM, there is one additional layer containing the F-value of the causal relationship of humidity, pressure, temperature, and wind speed with energy consumption/demand. This additional layer is also trained with backpropagation.

For training and validating both the models, 26 months of hourly data were used, and for the testing, the remaining three months of data were used. Hyndman and Koehler (2006) and Davydenko and Fildes (2013) have provided overviews of the metrics that could be used to evaluate the forecasts. In this contribution, the following three error metrics are used:

**Mean Absolute Error (MAE)**: this is the mean of the absolute difference between the original and the predicted values.

$$ MAE = \frac{1}{n} \sum \left| {e\left( t \right)} \right| $$

**Root Mean Squared Error (RMSE)**: the is the square root of the mean of the squared difference between the original and the predicted values.

$$ RMSE = \sqrt {\frac{1}{n} \sum e\left( t \right)^{2} } $$

**Mean Absolute Percentage Error (MAPE)**: this is the mean of the absolute error when the error is reported as a ratio of the original values.

$$ MAPE = \frac{100}{n} \sum \frac{{\left| {e\left( t \right)} \right|}}{o\left( t \right)} $$

where

$$ e\left( t \right) = orig\left( t \right) - pred\left( t \right) $$

In all the above formulae, *e(t)* is the error for time *t*, orig(t) is the original value at time *t*, pred(t) is the predicted value at time *t*, and *n* is the number of data points used in the test set. Among the three metrics, MAE and RMSE are the two most user error metrics. But they are scale-dependent, which indicates a requirement for an additional scale-invariant evaluation metric. Therefore, the MAPE is used. MAPE is also good for comparing the error rates among different datasets because of its scale-invariant nature. In this paper, only the performances of the bidirectional-multivariate-LSTM and the causal-LSTM (which by extension is also bidirectional and multivariate) are compared.

## Results

### Simulation results

First of all, the results with simulated data will be presented. The purpose of this set of results is to show that if there are two-time series where one time series is perfectly causing the other time series, which of the two LSTM architectures (Bi-LSTM or Entangled-LSTM) would perform better. For this purpose, two time series were generated, where the time series one is perfectly causing the time series two, and the task is to forecast the values for the second time series. Next, both the LSTM architectures were trained and tested for the simulated dataset. Table 1 contains the outcome of the comparison. The entangled LSTM clearly outperforms the Bidirectional LSTM. This shows the theoretical confirmation of the proposed method. That is, adding the causal information in perfect causal conditions will provide better forecasting than the model without the causal information. It is clear that when one-time series "perfectly" causes another time series, the performance of Entangled LSTM is better than the Bi-directional LSTM.

**Table 1:** The testing performance from the two LSTM architectures for the simulated data where the causality is established

Bidirectional LSTM				Entangled LSTM			
Dataset	MAE	RMSE	MAPE	Dataset	MAE	RMSE	MAPE
Simulated	0.15	0.12	0.08	Simulated	0.09	0.06	0.02

The MAE and RMSE are scale-dependent, and the MAPE is scale-independent. Therefore, for the purpose of understandability, the time series were normalized between 0 and 1

### Comparison of multivariate-bidirectional and multivariate entangled LSTMs

**Fig. 3:**
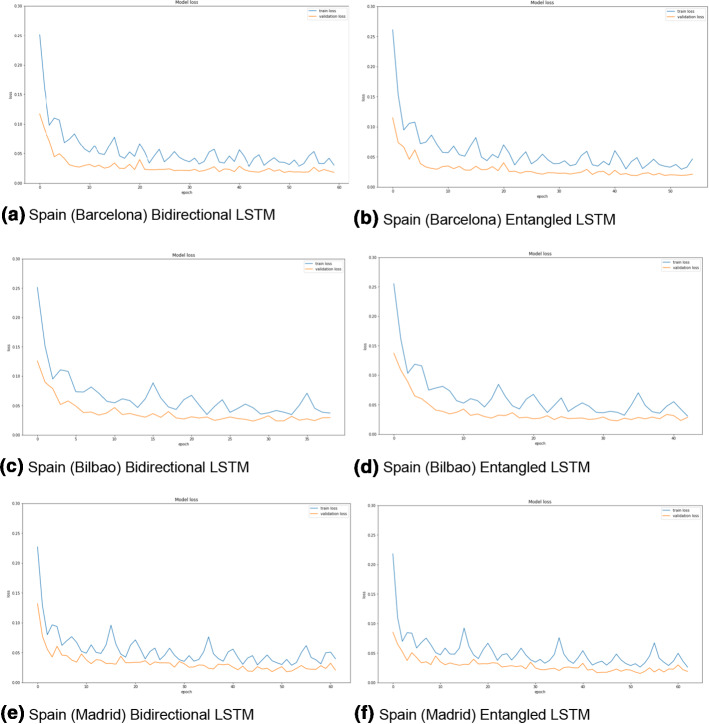

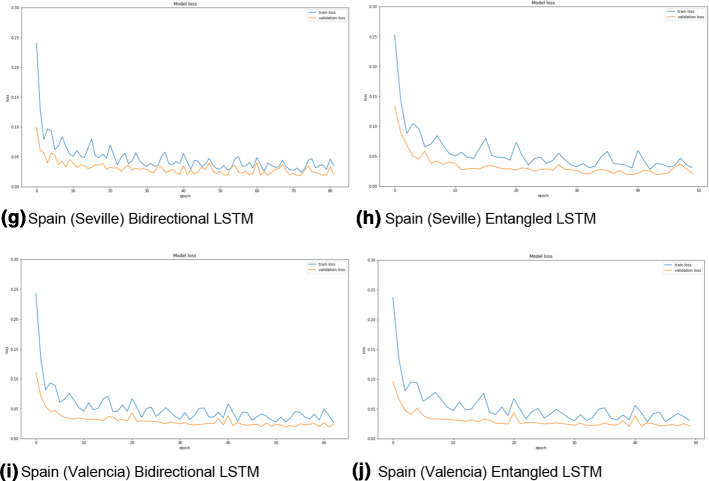
The training (blue curves) and validation (orange curves) losses from the first dataset (Spain dataset and the five sub-datasets). (Color figure online)

**Fig. 4:**
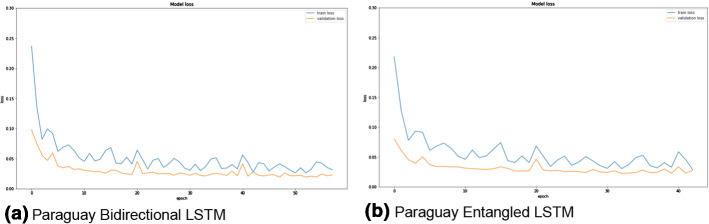
The training (blue curves) and validation (orange curves) losses from the first dataset (Paraguay dataset). (Color figure online)

**Fig. 5:**
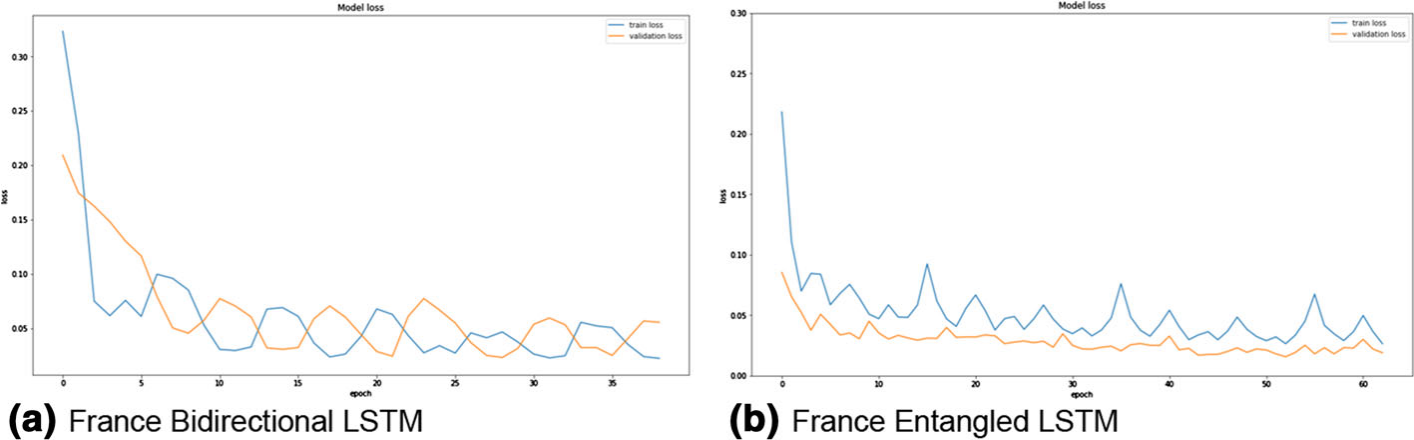
The training (blue curves) and validation (orange curves) losses from the third dataset (France dataset). (Color figure online)

The two LSTM architectures (bidirectional and entangled) are compared for the three datasets based on the different metrics (MAE, RMSE, MAPE). Figures 3, 4, and 5 show the training and validation losses for Spain (different sub-datasets based on the cities), Paraguay, and France datasets. It can be clearly observed from the losses that both the architectures are not overfitting on any of the datasets. Moreover, the training losses (the blue curves in all the figures) fluctuate in the range of 0.05 to 0.10 before eventually stabilizing. On the other hand, the validation losses (the orange curves in all the figures) fluctuate in a smaller range (0.02 to 0.04) and stabilize. None of the plots (in Figs. 3, 4, and 5) have any alarming differences between the training and validation losses. Therefore, it can be concluded that there was no overfitting of the data in any of the three cases.

As shown the Table 2, the entangled LSTM outperforms the bidirectional LSTM for all three datasets. The minimum improvement is for the Paraguay dataset (MAPE difference is 2.07%), and the maximum improvement is for the France dataset (MAPE difference is 7.73%). The highest difference between the two architectures is for the France dataset. This could be because of the reason that in the France dataset, the weather information is the most accurate for the geographical location. Therefore, the causality computed between the weather indicators (pressure, temperature, humidity, wind speed) and the energy consumption of the building (the France dataset has the data from an individual building) captures the causal relation that is closest to the reality among all the datasets. On the other hand, the Paraguay dataset having the minimum improvement shows that the data from the single weather station in the whole region does not exemplify the weather conditions that are in general covered by the 14 substations from which the data was collected. Another reason for the Paraguay dataset corresponding to the minimum improvement could be the original frequency of the weather parameters. In the Paraguay dataset, the original weather parameters were recorded every three hours, and the weather data was extrapolated to obtain the hourly frequencies for all the four weather parameters used in the paper. This could be the reason why the variability in the weather data in the Paraguay dataset was lower than in the other two datasets, and therefore, the causation with the energy data was not as informative as it was in the other two cases.

**Table 2:** The testing performance from the two LSTM architectures. As it is evident that the MAE and RMSE are scale-dependent, and the MAPE is scale-independent

Bidirectional LSTM				Entangled LSTM			
Dataset	MAE	RMSE	MAPE	Dataset	MAE	RMSE	MAPE
Spain (Barcelona)	3358.60	3929.88	12.55	Spain (Barcelona)	2692.17	3317.99	10.14
Spain (Bilbao)	3278.86	3873.20	12.49	Spain (Bilbao)	2462.59	2840.11	9.84
Spain (Madrid)	3583.12	4243.87	13.31	Spain (Madrid)	2848.33	3252.92	10.45
Spain (Seville)	3472.90	3917.47	12.89	Spain (Seville)	2283.42	2708.95	8.83
Spain (Valencia)	3885.11	4523.322	15.05	Spain (Valencia)	2117.02	2473.69	8.14
Paraguay	2651.78	3099.92	10.36	Paraguay	2101.28	2798.55	8.29
France	1962.67	2311.56	17.80	France	1066.35	1454.34	10.07

Another important aspect that can be observed is the differences between the bidirectional LSTM and entangled LSTM for the Sub-datasets of the Spain dataset. These sub-datasets are treated as independent datasets, and indeed there is no specific trend revealed from the presented forecasting and analysis. The order of the cities in terms of size of the population is Madrid, Barcelona, Valencia, Seville, and Bilbao, while their respective improvements from bidirectional to entangled LSTM are 2.86%, 2.41%, 6.91%, 4.04%, 2.65%. As a post-hoc prediction, the energy consumption data was divided in a way that the proportion of the energy consumed reflected the ratio of these cities' populations to the Spanish population. There was no significant difference in the results with either of the two methods. This shows that including the causal information is even more important in cases like the Spain dataset. In the following, an explanation is provided. Looking at the MAPE of the two methods, Valencia has the worst forecasting performance, and Bilbao has the best MAPE, looking at the bidirectional LSTM. This would indicate that adding the causal information plays a role in improving the forecasts. The MAPE for Valencia with entangled LSTM was cut down to half of what it was with bidirectional LSTM, while the MAPE for Bilbao was also reduced by almost a quarter with entangled LSTM.

In a nutshell, it is evident that adding the causal information for forecasting the energy consumption/demand improves the forecasting accuracy. This is shown across all three datasets. The range in MAPE for the entangled LSTM is 2.31% which, considering the variation in the three datasets, is neither alarming nor significant, especially because the three datasets cover large variations in terms of their area scope. As aforementioned, the Spain dataset covers national consumption while Paraguay and the France dataset cover regional and individual building consumption, respectively. This, combined With the proof that none of the models have overfitted in the training and validation phases, the generalizability of this method can be assumed.

### Comparison of univariate-bidirectional and univariate entangled LSTMs

The results from the previous multivariate forecasting show that using Granger's definition of causality and modelling the causal relationship between the two-time series can provide better forecasting results than simply using one or more time series to forecast another time series. Next, the comparison of univariate forecasting using bidirectional and Entangled LSTMs, was performed. For comparing the univariate forecasting quality, only MAPE was used because the other two metrics (i.e., MAE and RMSE) are scale-dependent and will follow the same trend as the MAPE. There are two key aspects that can be observed in Table 3. First, the univariate results are underperforming when compared to the multivariate results. This depicts the importance of considering the multiple weather features. Second, for all the univariate results, the Entangled LSTM outperforms the Bidirectional LSTM. This is another proof that incorporating causal relation between the weather features and the energy consumption is beneficial for the forecasting of energy consumption. From the univariate forecasting, it is also clear that the temperature is the most important weather feature for forecasting. Using the temperature, in both the Entangled and Bidirectional LSTMs, the MAPE is the lowest for all the datasets (and data-subsets). In some cases, temperature features marginally outperform all the other features; however, it emerges as the best feature to be used in the forecasting, nonetheless. In summary, With these univariate forecasting results, it is shown that it is important not only to include multivariability in the LSTM but also causal relationships.

**Table 3:** The univariate testing performance, MAPE, from the two LSTM architectures using temperature, humidity, wind speed, and pressure, separately

	Bidirectional LSTM				Entangled LSTM			
Dataset	Temp	Humid	WS	Pres	Temp	Humid	WS	Pres
Spain (Barcelona)	11.25	16.87	17.76	14.22	10.10	15.85	17.76	12.62
Spain (Bilbao)	14.07	18.70	13.46	14.28	11.45	13.63	13.46	12.37
Spain (Madrid)	15.19	18.10	15.36	16.39	13.23	14.49	15.36	14.79
Spain (Seville)	14.41	15.90	19.14	15.44	12.02	12.41	19.14	12.08
Spain (Valencia)	15.76	16.23	18.03	17.29	11.84	14.25	18.03	12.18
Paraguay	14.03	16.51	16.80	14.96	16.18	15.48	16.80	17.83
France	20.16	21.24	22.98	20.45	20.07	20.62	22.98	22.78

### Comparing early predictions

**Fig. 6:**
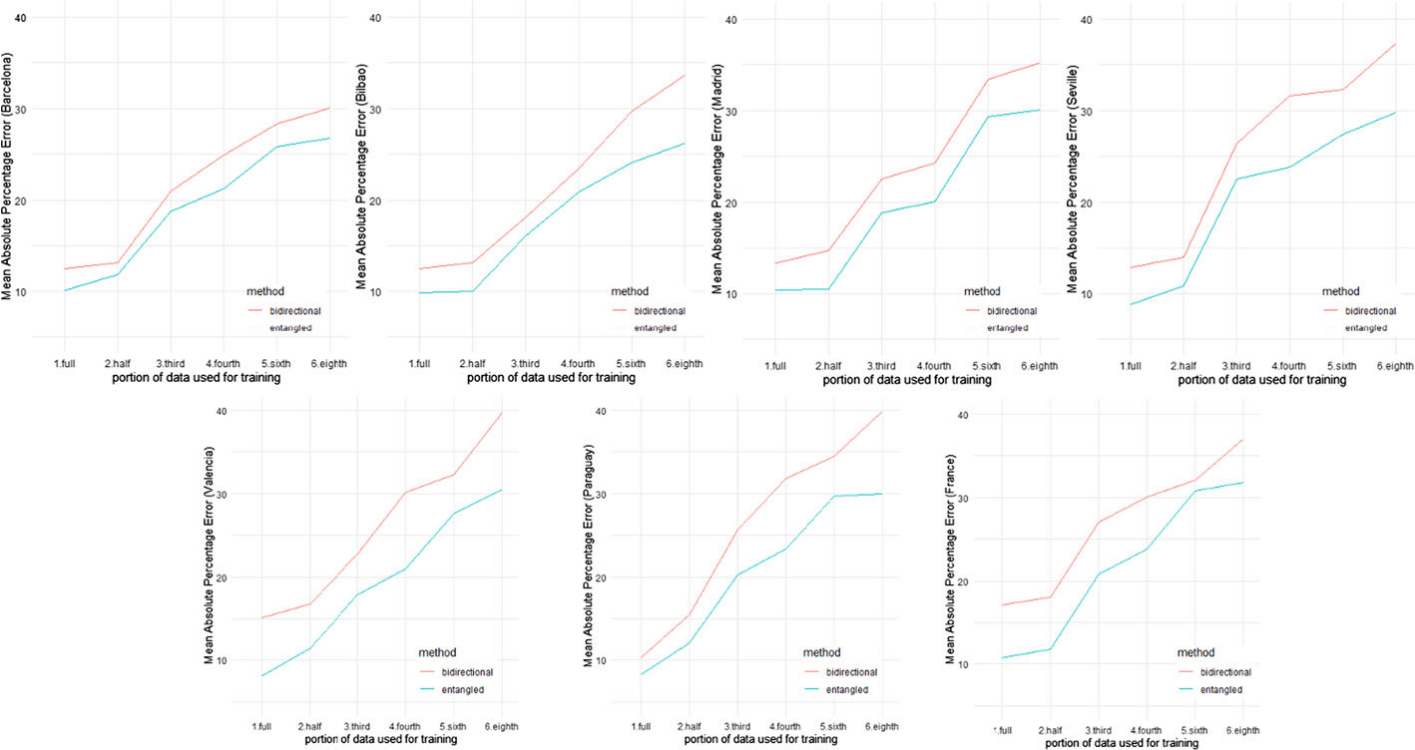
Results (MAPE values) for the early prediction experiments

While the Entangled LSTM outperforms the Bidirectional LSTM in all the comparisons, it is also important to understand "how much data is needed to obtain reliable forecasts?” To answer this question, less data was used to forecast than what was available in the given datasets. For example, the results in Sect. 4.2 are based on training the algorithms using the data from 29 months. In different experiments, the same forecasts were obtained using half data (14.5 months), third data (9.67 months), fourth data (7.25 months), sixth data (4.85 months), and eighth data (3.63 months). The purpose was to know at what proportion of the data the forecasting accuracy decreases to a level, as compared to the results from Sect. 4.2, that it ceases to be potentially useful. In all these cases, both the entangled and bidirectional LSTMs were compared. These results were also compared using the MAPE values of the forecasting performance. Figure 6 shows the results. In all the cases (i.e., all the datasets and the data subsets), it is observed that when the data is reduced from half of the original training data length to a third of the data length, a considerable increment in the MAPE occurs. Moreover, with subsequent reductions in the data, further increments in the MAPE values can be seen for all the datasets. In all these experiments, there are two key takeaways. First, as aforementioned, given these three datasets, half of the dataset (14.5 months) is sufficient to obtain similar forecasting performance as with the full data (29 months). Second, in early predictions, as is seen with univariate and multivariate forecasting, the entangled LSTM outperforms the bidirectional LSTM. Another proof for the initial hypothesis is that incorporating the causal information in the deep network for forecasting the energy demand yields better results than simply using the weather features in a multivariate LSTM.

### Comparing generalizability across datasets

Finally, the two algorithms are compared on the scale of generalizability. To perform such a comparison, cross-training testing was used. In other words, the training of the forecasting algorithm was carried out using one dataset, and the trained model was tested on another dataset. For example, training on the Spain dataset (all cities combined) and testing on France and Paraguay datasets. In this manner, both the algorithms were tested if they could use the learned model to predict in an unseen environment. The algorithm is deemed to be pseudo-generalizable if the testing accuracy on another dataset is comparable to the testing accuracy on the same dataset. For example, in the case of training on Spain and testing on Paraguay datasets, it can be observed from Table 4 that both LSTM models produce similar MAPE as when they were tested on the Spain dataset. When Bidirectional LSTM is trained on the Spain dataset, the testing MAPE for the Spain dataset is 13.32, while on the Paraguay dataset, the MAPE was 16.21. On the other hand, when the entangled LSTM is trained on the Spain dataset, the testing MAPE for the Spain dataset is 9.28, while on the Paraguay dataset, the MAPE is 11.21. In both cases, the algorithms can be considered generalizable. This difference is greater in the opposite case (i.e., when the training is done on the Paraguay dataset). However, the Entangled LSTM seems to be more generalizable than the Bidirectional LSTM. In the end, both algorithms do not seem to generalize for the France dataset. It is shown that when the algorithms are trained using the country-wide dataset (Spain), they generalize on the region-based dataset (Paraguay) but not on a building-specific dataset (France). Moreover, the region-based dataset does not generalize for the building-based dataset; also, the algorithms do not generalize or generalize to a smaller extent when they are trained on a smaller area and tested on a larger area (i.e., trained on region or building based data and tested on country or region based data, respectively). These results can be explained by the fact that the larger the area in the trained dataset, the higher the variance in the model, and therefore they generalize on specific cases, but the opposite is not possible. Another aspect worth mentioning about the results from Table 4 is that the MAPE differences are lower in Entangled LSTM than those for the bidirectional LSTM. This can be explained based on the fact that the causal relationship between the weather and the energy consumption across countries might be more generalizable than the temporal nature of the weather conditions across different countries. This might be why the Entangle LSTM achieves better generalizability than the Bidirectional LSTM.

**Table 4:** Results (MAPE values) from cross-training testing (i.e., training on one dataset and testing on another one)

Bidirectional LSTM	Testing dataset		
	Spain	Paraguay	France
Training dataset			
Spain	13.32	16.21	23.91
Paraguay	16.32	10.36	24.53
France	26.23	26.56	17.80

Entangled LSTM	Testing dataset		
	Spain	Paraguay	France
Training dataset			
Spain	9.28	11.12	18.93
Paraguay	11.92	8.29	19.21
France	21.53	19.24	10.07

## Discussion

### Implications for theory

Causal analysis has lately become one of the major and important components for improving modern processes, and a few examples include Capability Maturity Model Integration (CMMI), Six Sigma, and Lean. Incorporating the causal analysis is also becoming more important for the organizations than before to obtain high levels of process maturation. This is evident because of two facts. First, CMMI has assigned the Causal Analysis and Resolution at the Maturity Level five. Second, Six Sigma programs also often embed causal analysis in a curriculum that is of more challenging statistical levels. Identifying causal relationships in the data helps the systems to predict future events in a more robust manner than those done using just the correlations and regressions (Kleinberg, 2013). One of the key goals of causal analysis is to aid organizations in making better decisions (Zheng et al., 2020). Causal relations can provide better support in decision-making due to the following two reasons. First, by analysing causalities in a particular data set, one can not only gain a deeper understanding of the relationship between pairs of time series and provide better predictions, but one can also analyse the effects of certain past events or decisions on the future predictions and outcomes. Second, based on the known causal relations, one can forecast the effect before it takes place and simulates the effects of certain decision-making activities. Encoding the intricacies of the relationships in the given datasets using the causal analysis might be the root cause of the results that were obtained. This contribution shows how to improve the time series forecasting using by adding the causal information (entangled LSTM) to the basic forecasting model (bidirectional LSTM).

Furthermore, it is also backed by a few theoretical frameworks that the causal information should be used in the decision making. One of the most prominent ones is the Expected Utility Theory (von Neumann & Morgenstern, 1944; Savage, 1954). This theory has two assumptions, and incorporating the causal relation between the weather information and the energy demands shows how these two assumptions are satisfied. Especially because the results show an improvement in forecasting capabilities when the casual information is incorporated into the deep learning network as compared to when the causal information is absent from the forecasting problem. The first assumption is that each outcome has a corresponding utility to the decision-maker. In the presented case, this is the amount of energy consumed. The second assumption is that each outcome is assigned a probability. That is, each outcome is uncertain because there is always a lack of knowledge and evidence for an outcome to take place given a particular event or action. The theory also dictates that the outcome should maximize the expected utility. This lack of knowledge can be taken care of by using the causal relationship between the events and the outcomes. In this case, the energy consumption is the outcome, and the weather information creates the event. Therefore, it is intuitive to consider the causal relationship between energy consumption and weather data to effectively predict energy consumption. The results confirm the theory, where the entangled LSTM produces better forecasts than the bidirectional LSTM in all the cases, by showing that the power of causality in the predictor makes up for the lack of knowledge about the relation between an event (weather condition) and outcome (energy consumption). The inclusion of causal information is also supported by the causal decision theory (Joyce, 1999; Lewis, 1981; Maher, 1987; Nozick, 1993; Skyrms, 1982), which extends the Expected Utility Theory by dictating that knowing the outcomes of one must be aware of the causal relationship between an event and outcomes.

### Implications for practice

Deep learning architectures encode the representation of the input data at multiple levels of abstraction using their multiple processing layers (LeCun et al., 2015). These encodings then can and are used to generate better predictions and forecasts than the other basic machine learning and statistical methods (Husna et al., 2021; Kilimci et al., 2019; Xu & He, 2020). It has been shown that predictive analysis brings competitive advantages to organizations (Ransbotham et al., 2017), and by extension, using deep learning to obtain better predictions can improve the advantageous positions for these organizations. Most of the predictions and/or forecasts aid in the decision making for different operations such as supply chain management (Husna et al., 2021; Pacella & Papadia, 2021; Punia et al., 2020), digital marketing (Pan & Zhou, 2020; Qi et al., 2019), and financial decision making (Abu-Mostafa & Atiya, 1996; Geng et al., 2015; Kim et al., 2020a, 2020b; Xu & He, 2020). With the current availability of the data in huge quantities, traditional machine learning algorithms tend to saturate their training and risk overfitting and specificity to one case. On the other hand, because of their complex structures and multiple weights to be trained, deep learning architectures can handle this large amount of data in a manner that is beneficial for various operations in organizations. Another advantage of deep learning algorithms is that they do not need extensive pre-processing and feature engineering because such algorithms are known to function well with noisy and unstructured or semi-structured datasets. Most of the contributions cited in the related work section of this paper do not use many pre-processing and/or feature engineering schemes. This also gives an additional advantage to the organizations in faster decision-making. Two other virtues of deep learning algorithms that help organizations to invest less time in data-driven decision-making are: 1) the less requirement of human intervention during the training phase of deep learning algorithms, and 2) the support for parallel and distributed processing. The first one refers to the self-learning capabilities of the deep learning algorithms using the error-backpropagation through its multiple layers and therefore requiring less human intervention as compared to the traditional machine learning algorithms. Moreover, the second advantage is mostly due to the advancements in computing technology that allows the deep networks to be trained at scale and thus proving to be a big aid in fast data-driven decision support systems. With this contribution, by improving upon the state-of-the-art bidirectional LSTM networks mainly because of the use of causal information, the case of using deep learning architectures for operations research is emphasized.

The terms of the presented results (MAPE from entangled-LSTM), which are in the range of 0.08 and 0.11, stand comparable to some of the state-of-the-art contributions (Hrnjica & Mehr, 2020 report in the range 0.06—0.11 and Al Khafaf et al., 2019 report an average MAPE of 0.16); and better than others (Ishaq & Kwon, 2021: 0.35 and Somu & Ramamritham, 2021: 0.26); only Real et al. (2020) have reported a better MAPE range of 0.02—0.06. Considering that the better prediction of future energy consumption might lead to improving energy management processes and systems, this contribution also has certain implications for energy management systems. Efficient energy management is becoming a necessary process both at the supplier (i.e., smart grids) and consumer levels (i.e., energy management in smart homes). A smart grid energy management system contains multiple modules, among which the load and demand forecasting modules are also included (Chen et al., 2011). Effective forecasting systems can also support the real-time energy management systems in creating efficient load-balancing, operating routines, and minimizing operational costs (Luna et al., 2017). Moreover, better energy consumption forecasting can optimize the peak shavings for the utility grids and maximize the revenue for the grid (Shen et al., 2016). Finally, using highly accurate forecasts, it could be possible to minimize the power peaks and fluctuations while the grids are exchanging energy with each other or with the main grid (Arcos-Aviles et al., 2017). In terms of the consumer side of the energy supply chain, better energy forecasts can lead to better planning for optimizing smart home appliances (Hossen et al., 2018). Better energy consumption forecasts can have a major impact on the home energy management systems, which in turn can have a huge impact on the energy conservation, reliability, economics, and efficiency of the energy usage (Zhou et al., 2016). Whenever there is an option to choose between more than one source of energy, an efficient and individualized energy consumption forecast can also enable smart home energy management to switch among the multiple energy sources in a cost-effective manner (Olatomiwa et al., 2016).

### Limitations and future work

Our contribution extends state the art in energy demand forecasting by incorporating the causal relationships between the weather parameters and the energy consumption at three different levels, that is, country, a specific region, and individual building. However, there were certain issues that limited the extent of this work and simultaneously opened new avenues for exploration. For example, in this paper, all the causal information that was available in the data was added, that is, the pressure causes consumption/demand, the humidity causes consumption/demand, the temperature causes consumption/demand, and the wind speed causes consumption/demand. Although each of these causalities seems intuitive to be added to the forecasting model, not all might have the same amount of mutual information with the actual demand. Therefore, it is important to explore which ones or which combinations would provide the most appropriate amount of information for the desired increase in the forecasting capability. Furthermore, the datasets in Spain and Paraguay were limited by the amount of information provided. For example, the Spain dataset had the national energy demand, but the weather information was about the five largest cities in the country. It is safe to assume that the five largest cities can control a big proportion of energy consumed. However, it is a limitation in terms of analyses performed in this paper. On the other hand, in the Paraguay datasets, this problem was inverted. That is, local energy consumption data was available, but the weather information was centralised. Once again, it is not a completely valid assumption that the weather parameters would remain the same across a big region, but it is safe to assume that feeders in the high to medium vicinity of the weather station would be parameterised better by the causal relationship in the aggregate energy consumption data. In the future, it should be aimed to obtain the data such that the geographical spread of the two data streams is better matched than the two datasets. Different sources of energy (available in the Spain dataset) and the different modalities of usage (available in the France dataset) were not considered, where the causal relationship among each of these could have with the weather indicators. This choice was made to have one common analysis across the three datasets to showcase the generalizability of the proposed approach. However, exploring the different causal relationships between various energy usages and modalities with the weather conditions might also provide a better forecast for individual cases. Finally, for consistency of analysis across the three datasets, only four parameters were used to indicate weather conditions, that is, humidity, pressure, temperature, and wind speed. In the future, rainfall, snowfall, and wind direction, among other additional weather parameters, can also be considered.

From the current results presented in this paper, several venues emerge that could bring novel knowledge in the field of forecasting energy demand or consumption. First, at the forecasting level, exploring the multivariate nature of the causal relationships between the weather conditions and the energy consumption could improve the forecasting performance; because the interaction effects between the weather conditions would also be exploited in such a manner. Second, on a higher level, implementing and controlling the energy production using such methods (on a small scale) would provide an opportunity to study the effectiveness of the forecasting algorithm in the real-life scenarios because, with the current contribution, the practical nature of such an improved consumption forecasting could not be studied. Third, as it is with any dep learning application, the transparency and explainability of the algorithms are not up to the standards in some other industry sectors; therefore, after knowing that causality plays a significant role in the forecasting processes, the explanation for "how the forecasting works" could be provided to users at various levels, such as customers, managers, and policymakers.

## Conclusions

In a nutshell, this paper presents a deep learning method to forecast energy consumption using not only the weather data but also the causal relationship between the weather indicators and the energy consumption. For the casual modelling, the definition of causality between the two-time series provided by Granger was used. This method was applied to three freely available datasets and showed that in all the cases, that is, a country, a specific region, and an individual building, augmenting the forecasting model by the causal information also augments the forecasting performance. This contribution extends the state-of-the-art in four ways.

1. This paper proposes the inclusion of causal relations in the deep learning frameworks for forecasting the energy demand/consumption.
2. Extending the LSTM architecture by using the causal information about how weather conditions cause the changes in the energy consumption provides better forecasting results.
3. Using the causal relations within the LSTM framework also provides better early prediction. That is, it requires less amount of data to achieve a similar level of forecasting performance as it would have been required by a setup without the casual information.
4. By using cross-training–testing routines, this paper also shows the higher generalizability of the proposed method than the contemporary methods.

The theoretical and practical implications of the results are also provided, both of which indicate that including the causal information does not only confirm certain widely accepted theoretical frameworks but also provides better energy management opportunities both at the supplier (i.e., smart grids) and the consumer (i.e., smart homes) levels.

## Footnotes

1 https://www.kaggle.com/nicholasjhana/energy-consumption-generation-prices-and-weather.

2 https://data.mendeley.com/datasets/hzfwzzsk8f/4.

3 https://zenodo.org/record/3445332#.Ybcj6n3MJ24.
